# Dysregulation of β-Cell Proliferation in Diabetes: Possibilities of Combination Therapy in the Development of a Comprehensive Treatment

**DOI:** 10.3390/biomedicines10020472

**Published:** 2022-02-17

**Authors:** Natsuki Eguchi, Arvin John Toribio, Michael Alexander, Ivana Xu, David Lee Whaley, Luis F. Hernandez, Donald Dafoe, Hirohito Ichii

**Affiliations:** Department of Surgery, University of California, Irvine, CA 92697, USA; neguchi@hs.uci.edu (N.E.); atoribi1@uci.edu (A.J.T.); michaela@hs.uci.edu (M.A.); ivanax@uci.edu (I.X.); whaleyd@uci.edu (D.L.W.); luisfh2@uci.edu (L.F.H.); ddafoe@hs.uci.edu (D.D.)

**Keywords:** pancreatic β-cells, proliferation, antioxidative therapy, immunosuppression, diabetes

## Abstract

Diabetes mellitus (DM) is a metabolic disorder characterized by chronic hyperglycemia as a result of insufficient insulin levels and/or impaired function as a result of autoimmune destruction or insulin resistance. While Type 1 DM (T1DM) and Type 2 DM (T2DM) occur through different pathological processes, both result in β-cell destruction and/or dysfunction, which ultimately lead to insufficient β-cell mass to maintain normoglycemia. Therefore, therapeutic agents capable of inducing β-cell proliferation is crucial in treating and reversing diabetes; unfortunately, adult human β-cell proliferation has been shown to be very limited (~0.2% of β-cells/24 h) and poorly responsive to many mitogens. Furthermore, diabetogenic insults result in damage to β cells, making it ever more difficult to induce proliferation. In this review, we discuss β-cell mass/proliferation pathways dysregulated in diabetes and current therapeutic agents studied to induce β-cell proliferation. Furthermore, we discuss possible combination therapies of proliferation agents with immunosuppressants and antioxidative therapy to improve overall long-term outcomes of diabetes.

## 1. Introduction

Diabetes Mellitus (DM) is a metabolic disorder characterized by chronic hyperglycemia that affects an estimated 34.2 million people in the United States [1]. Diabetes is commonly associated with a plethora of complications, including diabetic nephropathy, retinopathy, and cardiovascular disease, and thus, early diagnosis and management is crucial to improve outcomes. T1DM and T2DM are distinguished by different pathological processes that ultimately lead to insufficient insulin levels to maintain normoglycemia. While T1DM results from autoimmune destruction of pancreatic β cells, T2DM is found in patients with insulin resistance, which initially results in β-cell overdrive and increased insulin secretion, which eventually drives β-cell exhaustion. Current treatments for both T1DM and T2DM focus on increasing insulin levels by improving β-cell function and/or by administering exogenous insulin. However, exogenous insulin is not sufficient to prevent the progression of DM and only works to delay the onset of comorbidities associated with long-term DM. Therefore, current research focuses on identifying islet cell regeneration methods by inducing β-cell proliferation and/or transdifferentiation. Unfortunately, this approach has been met with several obstacles, primarily that adult human β-cell proliferation has been shown to be very limited (~0.2% of β cells/24 h) and poorly responsive to many mitogens [2]. Furthermore, diabetogenic insults result in the dysregulation of pathways modulating β-cell masses, making it ever more difficult to induce proliferation. This review first briefly discusses alterations in β-cell function and mass in DM, followed by β-cell mass/proliferation pathways dysregulated in DM and current therapeutic agents studied to induce β-cell proliferation. Lastly, we discuss possible combination therapies with proliferation agents to improve overall long-term outcomes of DM.

## 2. Diminished β-Cell Function and Mass in DM

Pancreatic β-cells are endocrine cells that modulate blood sugar levels mainly through secreting insulin under basal conditions in a pulsatile manner and when stimulated by high glucose exposure after a meal. In this section, we discuss the impact of diabetogenic insult on β-cell function and health.

### 2.1. Pancreatic β-Cell Function in T2DM

Oral glucose tolerance test (OGTT) is commonly used to measure β-cell function by measuring blood glucose pattern following glucose administration. T2DM and healthy patients display distinctive glucose curve patterns during OGTT; patients with T2DM commonly (67.8%) show a monophasic blood glucose curve, even under treatment with metformin, to a prevalence of 80.9% in pre-diabetic patients with impaired glucose tolerance, while normal glucose tolerant patients exhibit biphasic curves [3,4]. Insulin sensitivity (homeostasis model of insulin sensitivity, HOMA2-S) for T2DM patients are similar regardless of the shape of their OGTT curve and is lower than would be seen in a normal population [3,5]. However, β-cell function adjusted for insulin resistance was significantly to be lower in patients with a monophasic curve, compared with patients with normal biphasic response, suggesting that the altered OGTT curve seen in T2DM is primarily a result of impaired β-cell function [4].

One theory of how β-cell function becomes impaired in T2DM is as insulin sensitivity decreases; β-cell increases their insulin production to compensate [6]. In turn, hyperinsulinemia leads to endoplasmic reticulum stress response, oxidative stress, and accumulation of reactive oxygen species leading to β-cell death [7,8,9]. As glycemia increases, insulin secretion rates from the β-cells become less responsive to changes in the glucose level, especially compared with non-diabetic subjects [10]. It is still unknown whether this dysfunction is caused by a reduction in β-cell mass or by a decrease in glucose sensitivity [11]. The alternative theory is that β-cell impairment on the first phase of insulin secretion leads toward T2DM [12]. Pancreatic β-cells release insulin in a pulsatile manner [13]. Normally, autocrine action of insulin in β-cell increases the packing of mature insulin into granules for exocytosis (first-phase insulin response), followed by negative feedback that prevents continuous insulin secretion [14,15]. In β-cells treated with fatty acids to imitate T2DM, insulin granules with synaptotagmin-9 were lost [14]. This dysfunction impairs the β-cell resting period in DM, characterized by increased levels of pro-insulin, as there is less time for intracellular insulin processing to proceed normally [16]. The impairment of β-cell insulin pulsatility has a potential genetic background, where the offspring and relatives of T2DM patients exhibit impaired insulin pulsatility [17,18]. A plethora of genes have been associated with an increased risk of β-cell dysfunction, including TCF7L2, CDKAL1, HHEX, CDKNA/2B, IGF2BP2, SLC30A8, and JAZF1 (accurately reviewed in [19,20,21]).

### 2.2. Pancreatic β-Cell Damage and Death in DM

Diminishing β-cell mass in diabetes occurs primarily through three pathways: apoptosis, necrosis, autophagy, and potentially ferroptosis [22]. In T1D, macrophage-derived IL-1 cytokine was found to be a strong intermediary that increases inducible nitric oxide synthase (iNOS) and nitric oxide production in β-cell, leading to β-cell death [22]. In contrast, in T2D patients, first, increased apoptosis was found, accompanied by reduced β-cell replication [23]. Additionally, autophagy pathway is normally responsible for maintaining normal islet homeostasis, especially in response to a high fat diet [24]. However, in T2D, gene expression of the normal autophagy pathway was altered, leading to accumulation of cytoplasmic vacuoles and increased β-cell death. This damage was found to be reversible by metformin treatment [25]. Therefore, while loss of β-cell mass in T1DM results from autoimmune destruction, diabetogenic insults result in β-cell dysfunction and death in T2DM. In the following section, we discuss β-cell mass regulating pathways that have been found to be dysregulated in T2DM.

## 3. Dysregulation of Pathways Regulating β-Cell Mass in DM

**Figure 1:**
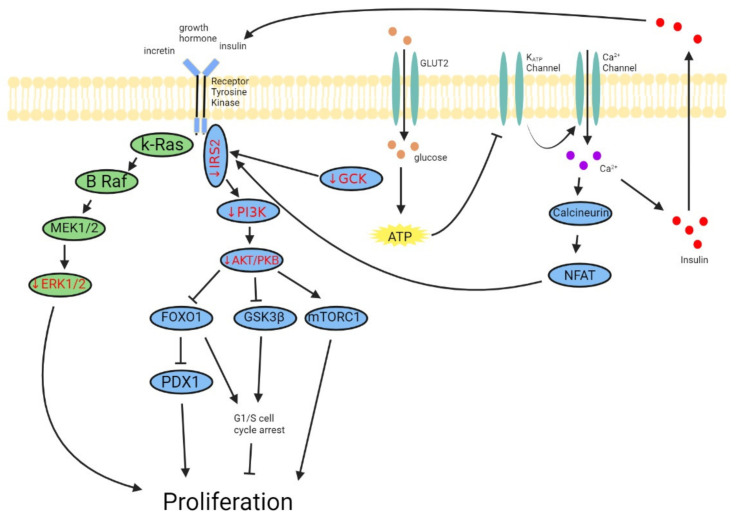
Pathways dysregulated in human T2DM islets and their potential downstream consequences (based on rodent and human studies). Downregulated genes found in T2DM human islets are indicated by the red color and arrow. T2DM β-cells exhibit alteration in gene expression of key upstream components of major pathways regulating β-cell mass, which may contribute to the reduced β-cell mass evident in T2DM patients.

Loss of β-cell mass in T1DM and T2DM occurs through distinctive methods. While T1DM results in β-cell mass loss through destruction by autoreactive immune cells, in T2DM, diabetogenic insults, most commonly hyperglycemia and hyperlipidemia, result in β-cell apoptosis, proliferation pathway dysregulation, and dedifferentiation. Stewart AF et al. have published a comprehensive review of proliferation pathways regulating human β-cell mass [26]. In this section, we focus on pathways regulating β-cell mass that have been shown to be dysregulated in T2DM conditions: PI3K-AKT/PKB pathway, Ras/Raf/Extracellular signal-regulated kinase (ERK) pathway, and cell cycle regulation (Figure 1).

### 3.1. PI3K-AKT/PKB Pathway

The PI3K-AKT/PKB pathway plays a crucial role in the regulation of β-cell function and proliferation through modulating insulin secretion and key proliferation genes including Forkhead box protein O1 (FOXO1), glycogen synthase kinase—3 (GSK3), and mammalian target rapamycin (mTOR) [27,28,29]. Importantly, islet cells of T2DM patients exhibit a significant reduction in AKT2 and a downward trend of PI3K expression [30]. Under physiological conditions, the AKT/PKB pathway is stimulated by insulin, growth factors, incretins, and glucose [31]. Both insulin and growth factors act through the stimulation of Insulin receptor substrate 2 (IRS2) receptors, which has been shown to play a central role in maintaining β-cell mass. IRS2-deficient β-cells in mice exhibit reduced proliferation and an inability to respond to external insulin stimulation, while overexpression of IRS2 receptors in β-cells induces proliferation in rats and decreases apoptosis in humans β-cells under hyperglycemic treatment in vitro [32,33]. Importantly, it has been demonstrated that islet cells of T2DM patients exhibit significantly lower the levels of IRS2 receptors compared with normal glucose tolerant patients [30,34]. Glucose metabolism also stimulates the AKT/PKB pathway through the IRS2 receptors. In addition to activating IRS2 receptors through stimulating insulin secretion, glucose metabolism also directly increases IRS2 receptor expression through glucokinase (GCK) activity and calcineurin/NFAT pathway [35,36,37]. GCK may be an important point of intervention as T2DM patients exhibit downregulation of GCK expression and a patient with a GCK mutation that resulted in 8.5 times higher affinity for glucose demonstrated significantly higher β-cell proliferation rates compared to control patients [30,38,39].

The inhibition of FOXO1 and GSK3β, and the activation of mTORC1 are important downstream targets of the AKT/PKB pathway. FOXO1 has been described as a double-edged sword as it has both protective and harmful effects. Under oxidative stress conditions, FOXO1 activates expression of Neuro D and MAF BZIP transcription factor A(MafA), two insulin 2 gene transcription factors important for β-cell identity and function [40]. On the other hand, the constitutive nuclear expression of FOXO1 prevents pancreatic and duodenal homeobox 1 (PDX-1) induced β-cell proliferation by downregulating expression of PDX-1 [41]. Furthermore, mice with IRSKO exhibit nuclear restriction of FOXO1 in β-cells, significantly reduced β-cell mass, and suffers from β-cell failure; however, the ablation of one allele of FOXO1 is sufficient to restore β-cell proliferation, indicating that FOXO1 is an important target of IRS2 for regulation of proliferation [41,42]. mTORC1 is another downstream target of the PI3K-AKT/PKB pathway that has been shown to be important in inducing β-cell proliferation. Counterintuitively however, it has been shown that islets of T2DM patients exhibit elevated mTORC1 levels [43]. In mice, forcing mTORC1 expression in islet cells significantly increases islet cell mass consistent with the consensus on the importance of mTORC1 on β-cell proliferation, but the islets demonstrated transcription pattern consistent of neonatal immature islets [44]. Supporting this study, Jia YF et al. showed that the treatment of T2DM patient islets with diabetogenic insults results in reduced β-cell proliferation secondary to upregulation of TBK1 and subsequent downregulation of mTORC1. Interestingly, the upregulation of mTORC1 through the inhibition of TBK1 in INS-1 832/13 β-cells augments β-cell proliferation while compromising the expression of function maintaining genes under basal conditions [45]. These results are in line with previous studies that showed an inverse relation between proliferative capacity and β-cell maturity [46]. Lastly, the inhibition of GSK3β through phosphorylation by AKT also plays an important role in maintaining β-cell mass. Active GSK3β phosphorylates Cyclin D2 and Cyclin D3 and causes G1/S cell cycle arrest, which is further discussed below [29]. Furthermore, activated GSK3β phosphorylates PDX-1, promoting its degradation by proteasomes [47]. Importantly, hyperglycemic treatment of human islets in vitro caused hyperactivation of GSK3β and subsequent increased phosphorylation and degradation of PDX-1 [47]. In addition to decreasing proliferation, the suppression of PDX-1 resulted in the downregulation of glucose transporter 2 (GLUT2); since this would result in decreased glucose metabolism, this may aggravate the reduced activation of PI3K-AKT/PKB pathway in DM [47]. Studies evaluating the expression levels of Glut2 in T2DM islets have yielded conflicted results; while some reported downregulation, others reported no change [30,38].

### 3.2. ERK1/2 Pathway

ERKs are one of the classical mitogen activated protein kinase (MAPK) signaling that has been extensively studied for their role in regulation of cell proliferation in pancreatic β-cells [26]. ERKs are commonly activated by growth factors, and its activation is mediated by MAPK3s (Raf isoforms) and MAP2Ks (MEK1/2 isoforms) [48]. The importance of ERK1/2 for β-cell proliferation has been demonstrated primarily in rodent models. Mek1- and Mek2-deficient mice with abrogation of ERK signaling in β-cells exhibit insufficient insulin production with lower β-cell proliferation and reduced β-cell mass [49]. Pharmacological agents such as Trefoil factor 2, genistein, and Epoxypukalide induced mice β-cell proliferation in vitro and in vivo through ERK1/2 activation, an effect abrogated with ERK1/2 inhibition [50,51,52]. Although fewer studies have been conducted in human islets, it has been shown that genistein also induces human β-cell proliferation through activation of ERK 1/2 pathway in vitro, and ERK 1/2 inhibition results in abrogation of this effect [51]. Furthermore, the inhibition of men1, inhibitor of k-Ras and downstream ERK1/2, stimulates human β-cell proliferation in vitro [53]. Importantly, pancreatic islets of T2DM patients exhibit significantly lower levels of phosphorylated ERK 1/2 [49]. Supporting this finding, human islets from type 2 DM donors were reported to be 80% deficient in the p21 (Cdc42/Rac)-activated kinase, PAK1, which has been shown to be important for activation of ERK1/2 amongst other things in both rodent and human [54]. Furthermore, HNFα-deficient mice islets exhibit impaired ERK 1/2 phosphorylation in response to EGF treatment, suggesting that HNFα is required for ERK1/2 activation; interestingly, T2DM islets also exhibit downregulation of HNF-α as well [30,55].

### 3.3. Altered Cell Cycle Dynamics

In addition to disruption of the PI3K-AKT/PKB and ERK1/2 pathways, alterations in cell cycle regulators may also play a part in reduced β-cell mass in T2DM. The difficulty of inducing human β-cell proliferation even under physiological conditions is well known, and this has been suggested to be partly due to the majority of G1/S proteins involved in cell cycle progression being expressed cytoplasmically rather than in the nucleus [56]. Compounding these issues, islets from T2DM patients exhibit elevated proliferating cell nuclear antigen (PCNA) expression with concomitant downregulation of cyclin dependent kinase 2 (CDK2) and p27-kip1, suggesting that diabetic islets from T2DM patients are able to enter the cell cycle but are unable to proliferate due to G1/S phase arrest [57]. p27-kip1 has dual effect in regulating cell cycle progression: (1) inhibition through inhibiting cyclin A/cyclin E/cdk1/cdk2) and (2) promotion through stimulating the nuclear translocation of CDK2/4 and Cyclin D [58]. However, it has not been evaluated whether Cyclin D and CDK2/4 nuclear expression is downregulated in T2DM islets, and thus, whether the downregulation of p27-kip1 has beneficial or harmful effects is unclear. On the other hand, the downregulation of CDK2 has clear consequences; pancreatic CDK2-deficient mice exhibit β-cell dysfunction and defects in β-cell proliferation [59]. Furthermore, in human islets, it has been shown that CDK2 binds to and phosphorylates FOXO1 in a glucose dependent manner, causing nuclear exclusion; thus, the downregulation of CDK2 would result in constitutive expression of FOXO1 in the nucleus and subsequent β-cell dysfunction [59]. Moreover, FOXO1 has been shown to downregulate cyclin D2, which plays an important role in G1 phase progression, feeding into a vicious cycle [60]. A possible explanation of the downregulation of CDK2 in T2DM is the downregulation of IRS receptors in T2DM patients. IR-deficient β-cells in mice exhibited high levels of nuclear FOXO1 with concomitant downregulation of CDK2; CDK4; and cyclin D2, D3, and E expression (CKD2: virtually absent, CDK4: ~85% reduction, cyclin D2 and D3: >80% reduction, cyclin E: 42% reduction in IR-deficient β-cell compared with the control) [57]. The expression of human insulin receptor B isoform in IR-deficient β cells in mice restored FOXO1 cytoplasmic expression and phosphorylation, and CDK2, CDK4, and cyclin E protein expression, further highlighting the importance of proper IRS expression and function in restoring β-cell masses [57]. Lastly, the overexpression of CDK6 and Cyclin D2 in human β-cells increased proliferation to 13% of β-cells from negligible levels in vitro [61,62,63]. Additionally, in vivo, transplanting 1500 islet equivalent (IEQ) of CDK6 and cyclinD2 overexpressing human islets into non-obese diabetic (NOD)-SCID mice yielded similar blood glucose and intraperitoneal glucose tolerance test (IPGTT) results to those transplanted with 4000 IEQ of untreated human islets, suggesting increased proliferation and function in vivo [61]. Thus, all in all, T2DM patients exhibit dysregulation of cell cycle regulators, and further studies must be conducted to evaluate the efficacy and safety of therapeutic agents targeted at stimulating cell cycle regulators.

## 4. Current Therapeutic Agents

Both T1DM and T2DM pathogeneses involve the loss of β-cell mass, and the identification of mitogenic factors that may stimulate β-cell proliferation is crucial in developing a therapeutic regimen to improve prognosis of DM. Unfortunately, adult human β-cell proliferation has been shown to be very limited (~0.2% of β-cells/24 h) and poorly responsive to many mitogens that have been shown to induce expansion in rodent models, including glucagon-like peptide 1 (GLP-1) analogs, IGF-1, and hepatocyte growth factors, to name a few [2]. Furthermore, recent studies have identified age-dependent factors in humans that influences the ability of β-cells to respond to specific mitogens; thus, juvenile and adult pancreatic β-cells require different mitogens for expansion. This section discusses therapeutic agents that have been shown to induce proliferation in juvenile and adult human pancreatic β-cells.

### 4.1. Therapeutic Agents for Juvenile Human Pancreatic β-Cells

#### 4.1.1. GLP-1 Analogs

GLP-1 analogs are a class of anti-diabetic medication currently approved for the treatment of T2DM. It has been shown to be effective in improving Hemoglobin A1C (HbA1C) by 0.6–1.5% over a three year period predominately through augmentation of glucose-stimulated insulin secretion [64]. While GLP-1 analogs have been shown to promote β-cell expansion in rodent models, prior studies investigating the effect of GLP-1 analogs on human β-cell proliferation have yielded conflicting results [65,66]. Although liraglutide, a GLP-1 agonist, indeed induced the proliferation of β-cells (0.042% vs. 0.082% control vs. liraglutide, *p* < 0.05) in an in vitro study, this increase would not be sufficient to serve as a treatment for DM [67]. This discrepancy may partly be a result of the age of donors. Dai C et al. recently demonstrated that exendin-4, a GLP1 analog, induced proliferation in juveniles (ages 0.5–9 years) through the activation of the calcineurin/nuclear factor of activated T cells (NFAT) pathway but not in adult human β-cells (ages 20 and up) [68]. The basal juvenile β-cell proliferation rate decreased with age and the increase in proliferation rate in response to exendin-4 treatment was inversely correlated with age.

#### 4.1.2. Prolactin

Prolactin is a hormone produced by the anterior pituitary gland. While it is most well-known for its function in lactation and homeostatic control, it has also been shown to play a crucial role in β-cell adaptation during pregnancy [69,70]. During pregnancy, β-cell mass is increased two- to threefold in rodent models and 40% in humans [71,72]. Furthermore, low prolactin levels in non-pregnant human models show a higher prevalence of DM and impaired glucose regulation [73]. Importantly however, the frequency of β-cell proliferation was not increased in pregnancy, as evidenced by the non-significant change in Ki67% insulin+ cells [72]. Instead of proliferation, it has been suggested that islet cell neogenesis may be responsible for the increased β-cell mass evident during pregnancy [72]. Supporting this finding, a study demonstrated that, while human recombinant prolactin treatment of human pancreatic β-cells increased in vitro survival by 37%, there was no apparent increase in proliferation [74]. Furthermore, while the unresponsiveness of β-cells to prolactin has partly been attributed to the fact that adult human β-cells express little to no prolactin receptors (PRLR), restoration of human PRLR on human β-cells rescued the JAK/STAT5 signaling pathway but failed to activate proliferation [71]. On the other hand, fetal pancreatic islets during late gestation express high levels of PRLR [75]. Currently, no studies have assessed the effect of prolactin on juvenile β-cell proliferation, and therefore, further studies in this field will be crucial to determine if prolactin could be used as therapy for early T1DM.

#### 4.1.3. PDGF

Platelet-derived growth factor (PDGF) is a serum growth factor that has been shown to be involved in β-cell proliferation. In a rodent model, a decrease in PDGF-AA serum produced by osteoblast cells in bones decreased β-cell proliferation in vitro [76]. The proliferative capacity of PDGF appears to be age dependent, however, as PDGF treatment increased β-cell proliferation in juvenile mice but not in adult mice. Similar effects were seen in human β-cells; PDGF treatment increased β-cell proliferation in juveniles through activation of ERK1/2 pathway but not in adults [77]. This is likely due to PDGF receptors not being expressed in adults as overexpression of human PDGFR-a in β-cells of adult transgenic mice increased β-cell proliferation [78]. Thus, PDGF may be a promising therapy to enhance β-cell proliferation in children with early onset T1DM.

#### 4.1.4. WISP1

Wnt-induced signaling protein 1 (WISP1) is a circulating factor involved in a wide range of tissue specific biological functions including cell growth, tumorigenesis, as well as β-cell proliferation in both rodents and humans. WISP1-deficient mice lead to reduced β-cell proliferation during the early postnatal period; in addition, injecting recombinant mouse Wisp1 in WISP 1-deficient mice showed a 1.7- to 2-fold increase in β-cell proliferation compared with saline treatment in both juvenile and adult mice [79]. Similarly, treatment of human islets (average age of 54.1 years) with recombinant human WISP1 increased β-cell proliferation up to 2% through the activation of the AKT/PKB pathway, evidenced by the abrogation of proliferative effects of WISP1 when rodent and human β-cells were co-treated with WISP1 and an AKT inhibitor. Importantly, children aged 2–5 years have been shown to have significantly higher circulating WISP1 levels compared with adults aged 28–45 years; thus, usage of young blood factors is a potential way to increase β-cell proliferation in adult humans [79]. However, further studies must be conducted to confirm safety of WISP1 treatment as it has a plethora of target sites throughout the body that may lead to pathological conditions including cancer.

### 4.2. Therapeutic Agents for Adult Human Pancreatic β-Cells

#### 4.2.1. Gastrin

The ability of gastrin to improve glycemic control in T2DM has been indirectly demonstrated by clinical trials that demonstrated improved HbA1C in patients receiving proton pump inhibitors (PPIs), which indirectly elevates serum gastrin levels [80,81,82]. Whether this is through improving β-cell function or altering β-cell mass is not known. Gastrin has been shown to be a potent inducer of rodent β-cell proliferation; however, studies evaluating the effect of gastrin on human β-cell proliferation have yielded conflicting results [83,84]. Consistent with the experimental data in rodents, gastrin treatment increased proliferation of human 1.1B4 β-cells in vitro [85]. Supporting this, Meier et al. reported high rates of β-cell proliferation adjacent to gastrinomas, gastrin producing tumors, in the adult human pancreas [86]. In contrast with this report, Bruer et al. reported that there was no difference in β-cell area and replication between DM patients receiving PPI treatment and those without [87]. Furthermore, in an in vitro study, while gastrin alone was able to increase survival of insulin + cells when human islets from healthy donors were incubated with gastrin, gastrin alone failed to increase the number of insulin+ cells [88]. However, interestingly, treatment of pancreatic cells in gastrin or gastrin + epidermal growth factor (EGF) increased the expression of β-cell transcription factors PDX-1 and Insulin in CD19+ pancreatic duct cells. Thus, it is possible that gastrin may induce β-cell neogenesis through transdifferentiation of pancreatic duct cells, an effect augmented to significant levels with cotreatment with EGF [88]. However, further studies must be conducted to elucidate this effect.

Gastrin may also contribute to β-cell mass through maintaining β-cell identity under diabetic conditions. For example, gastrin treatment of islets from DM patients resulted in increased expression of insulin (INS), PDX-1, MAFA, NKX homeobox 1 (NKX6.1), NK2 homeobox 2 (NKX2.2), monitor neuron and pancreas homeobox 1 (MNX1), and common β-cell markers [89]. Islets from patients with higher HbA1C, signifying higher average blood glucose level in the prior 3 months, experienced a more profound increase in these β-cell transcription factors compared with those with lower HbA1C [89]. This is possibly due to the increased gastrin receptor expression, cholecystokinin B receptor (CCKBR), in insulin positive cells from donors with higher HbA1C [89]. Interestingly, while PPI treatment of patients with HbA1C ≤ 7% only resulted in an average of 0.05% decrease in HbA1C, patients with HbA1C > 7% and HbA1C > 9% showed 0.5% and 1.2% reduction in HbA1C, respectively [82,90]. Thus, further studies elucidating the mechanism in which PPI and gastrin improve glycemic control may help in directing this treatment approach to the appropriate patient population. Lastly, while PPI benefited T2DM patients, a clinical trial that studied the efficacy of combination therapy with sitagliptin and lansoprazole(PPI) in patients with recent onset T1DM demonstrated no changes in C peptide levels and HbA1C levels between patients receiving PPI and those without [91]. Thus, while combination therapies with drugs that increase gastrin levels are promising avenues to improve glycemic control in T2DM, possibly through increasing β-cell mass, further studies must be conducted to discover novel combination therapies with gastrin to treat T1DM.

#### 4.2.2. DYRK1A Inhibitors

Small molecule inhibitors of dual specificity tyrosine phosphorylation regulated kinase 1A (DYRK1A) have gained widespread interest due to its potent ability to induce human β-cell proliferation. Among the known DYRK1A inhibitors that have been shown to induce human β-cell proliferation including aminopyrazines, thiadiazine, and 5-IT, harmine has been the most studied [92,93,94]. Harmine treatment in vitro of human pancreatic β-cells increased proliferation to approximately 1.3% from negligible levels through activation of DYRK1A -NFAT pathway, which has been shown to upregulate transcription of proliferation related genes including cell cycle regulators and IRS-2 receptors [37,95]. Similarly, when human islets were transplanted into the renal capsule of NOD-SCID mice, harmine treatment in vivo increased BrdU and Ki67 labeling in β-cells by 2 to 3 folds compared with saline treatment, and improved blood glucose levels and intraperitoneal glucose tolerance test [96]. Several combinations have been explored to improve the potency of harmine. First, harmine and TGFβ inhibitor combination resulted in increased Ki67 positive β-cells to 5–8% compared with the 1–3% in harmine alone in vitro [97]. Secondly, harmine and GLP1 combination treatment of human islets resulted in an average proliferation rate of 5%, similar to the combination with TGFβ. Importantly, blood glucose levels after transplantation of 1500 IEQ or 500 IEQ with harmine and exendin-4 treatment in streptozotocin (STZ) diabetic NOD scid gamma mouse (NSG) mice showed no significant difference, a result that was accompanied by a 3-fold increase in proliferation in the harmine + exendin-4 treatment arm compared with the saline group [66]. Furthermore, while the concern of increased proliferation in β-cell is the worry of naive phenotype, both harmine + TGFβ and harmine + GLP-1 analog treatment of normal and T2DM human islets resulted in an increased expression of key β-cell markers including PDX1, NKX6.1, MAFA, and MAFB [66,97]. With its potent ability to induce β-cell proliferation, DYRK1A inhibitors appear to be a promising regeneration treatment for both T1DM and T2DM; however, clinical utility is hampered by central nervous system off target effects. To curtail these effects, several derivatives of current inhibitors have been evaluated for increased kinase selectivity, and decreased off-target effects and cytotoxicity [93,98,99,100,101].

#### 4.2.3. GABA

The neurotransmitter γ-aminobutyric acid (GABA) is a signaling molecule secreted from β-cells. It has been reported to be important in insulin exocytosis and glucose stimulated insulin secretion in human β-cells, and GABA signaling has been shown to be dysregulated in β cells from T2DM patients [102]. In addition to its role in insulin regulation, GABA has also been shown to stimulate β-cell proliferation in both rodent and human β cells [103,104]. In a rodent model, GABA treatment prevented the development of DM and reversed DM through preservation and restoration of β-cell mass in STZ-induced NOD Mice [103]. The in vivo GABA treatment of NOD-Scid mice or STZ-induced C57BL/6J mice transplanted with human islets increased Ki67+ insulin+ islet cells and decreased apoptotic islet cells [104,105]. Purwana I et al. demonstrated that, in human β-cells, GABA evokes Ca^2+^ influx, which subsequently results in AKT and cAMP response element binding protein (CREB) phosphorylation, suggesting that GABA signals through the PI3K/AKT pathway. Furthermore, GABA treatment increased IRS-2 mRNA expression [105]. The therapeutic effects of GABA have been thought to be due to its ability to induce β-cell proliferation and to augment insulin secretion but also its anti-inflammatory and immunosuppressive properties. In rodent models, GABA treatment reduced circulating inflammatory cytokines including IL-1β, TNF-α, IFN-γ, and IL-12 in STZ-induced mice, and in vitro, GABA reduced CD4+ and CD8+ T cells and increased regulatory T cells [103]. Furthermore, the activation of GABA(A) receptor has been shown to inhibit proliferation in T cells isolated from human peripheral blood mononuclear cells in vitro [106]. Based on these compelling data, clinical trials with GABA have been initiated for prevention and treatment for new onset T1DM; however, its safety and efficacy has not been reported thus far [107]. Lastly, GABA has been shown to improve insulin resistance through increasing peripheral expression of Glut4, reducing inflammation, and decreasing blood glucose levels [108]. Oral treatment with GABA inhibited the high-fat-diet-induced glucose intolerance, insulin resistance, and obesity through its anti-inflammatory effects in C57BL/6J mice [109]. Thus, GABA is a promising treatment for both T1DM and T2DM, and further studies evaluating its efficacy and safety is warranted.

Several other agents such as GSK3β inhibitors, transforming growth factor β (TGFβ) inhibitors, IKKε and EBP1, and Serpin B1 have been evaluated for their ability to induce β-cell proliferation in human islets [110,111]. GSK 3β inhibitors, LiCl and 1-Akp, in combination with glucose, stimulated mTOR-dependent DNA synthesis, cell cycle progression, and proliferation of human β-cells [112]. Additionally, pharmacological agents with dual GSK3β and DYRK1A inhibition have been shown to increase β-cell proliferation to 3–6% from negligible levels in human islets [92,100]. However, whether the proliferated β-cells exhibit mature phenotype has not been evaluated yet. TGFβ inhibitors have also been shown to induce proliferation. Combination treatment in vitro with small molecule menin-MLL inhibitors and TGFβ inhibitors synergistically increased human β-cell proliferation through the downregulation of cell cycle inhibitors without affecting insulin production, suggesting sustained mature phenotype. Furthermore, in vivo, TGF-β inhibitors also successfully increased Ki67+ β-cells from approximately 0.1% to 0.5% in human islets transplanted in NSG mice [113]. TGF-β has also been tested in combination with DYRK1a as discussed earlier [114]. While promising, clinical utility of TG-β has been questioned due to its potential harmful effects to other organs at evaluated doses.

## 5. New Therapeutic Approach

In both T1DM and T2DM, β-cells face multi-prong challenges that limit their function, survival, and proliferative capacity. Therapeutic approaches solely focused on increasing β-cell proliferation are not sufficient in improving the long-term outcomes of DM. Combination therapy focused on protecting β-cells from apoptosis/dedifferentiation from diabetogenic insults in the case of T2DM or protecting islets from activated autoreactive immune cells for T1DM is crucial to developing a comprehensive approach. Here, in this section, we discuss potential combination therapy with nuclear factor erythroid factor 2 related factor 2 (Nrf2) activators and immunosuppressive medication to magnify the beneficial effects of β-cell proliferation agents in the treatment of T1DM and T2DM.

### 5.1. T2DM

T2DM is caused by a combination of two factors: (1) impaired insulin secretion and death of pancreatic β-cells and (2) insulin resistance. Thus, while β-cell proliferation is an attractive therapeutic approach in improving prognosis of T2DM, without targeting insulin resistance, β-cell proliferation may serve only as a temporary bandage. Unfortunately, current treatment options for insulin resistance are very limited and are primarily focused on weight management [115]. Worse yet, the chronic hyperglycemia resulting from insulin resistance results in β-cell dysfunction and death, promoting a vicious cycle. The effects of hyperglycemia on β-cells have been extensively studied, and more recently, oxidative stress has been highlighted as one of the major downstream consequences that has detrimental impact on β-cell function and survival possibly due to the limited antioxidative capacity of β-cells [116]. Compared with α-cells, β-cells have a significantly lower expression of catalase and glutathione peroxidase, and exposure to oxidative stress conditions results in significantly lower survival and viability of β-cells, reducing the β/α cell ratio [117]. Thus, combination therapy with therapeutic agents targeted at alleviating oxidative stress may improve outcomes through providing protection of newly proliferated β-cells.

Nrf2 activators hold tremendous potential to fulfill this role. The Nrf2 pathway is an important regulator of cellular defense against oxidants and in human pancreatic islets, controls the expression of key antioxidants including NAD(P)H: Quinone oxidoreductase, Heme oxygenase 1 (HO-1), glucose 6 phosphate dehydrogenase (G6Pd), sulfiredoxin-1, and thioredoxin reductase1 (TXNRD1) [118]. Pharmacological activation of Nrf2 pathway by dimethyl fumarate (DMF), oltipraz, dh404, curcumin, sulforaphane, vitexin in human and/or rodent β-cells have been shown to protect β-cells under different stressors, including glucolipotoxicity and oxidative stress, by preserving β-cell function and mass [118,119,120,121,122]. Furthermore, Nrf2 activation has been shown to be sufficient to drive human β-cell proliferation in vitro, supporting the beneficial effect of Nrf2 activation on proliferation [123,124]. Lastly, a cross sectional study that evaluated TNF-α, HO-1, and Nrf2 levels in β-cells of normal glucose tolerant, prediabetic and T2DM patients found that while TNF-α levels increased with progressing DM, HO-1 and Nrf2 levels decreased, indicating an impaired antioxidative system in DM conditions [34]. In addition to offering protection from diabetogenic insults, more recent studies have also elucidated the potential beneficial effect of Nrf2 activators on insulin resistance. Thus, in T2DM, elevating Nrf2 levels to improve β-cell function and survival while increasing β-cell mass through proliferation agents may be a novel holistic approach to improve long-term outcomes of T2DM.

### 5.2. T1DM

Several immunosuppressive regimen have been tested for the treatment of T1DM. Immunotherapy against new-onset T1DM is largely grouped into three categories: therapies targeting T cells, targeting B cells, and anti-inflammatories and cytokines. Of the therapies targeting T cells including cyclosporine and anti-thymocyte globulin, anti-CD3 monoclonal antibody(teplizumab) showed the most promising results, delaying the diagnosis by at least 2 years versus the placebo. However, 20 to 55% of teplizumab-treated participants developed antidrug antibodies, and thus, the long-term efficacy has not been established yet [125]. For B cell therapy, while rituximab improved HbA1C in T1DM patients, its effects were only seen temporarily as immune tolerance was not induced [126,127]. Anti-inflammatory therapy, IL-6Ra blockade recently showed no clinical efficacy while TNF-α blockade successfully delayed C-peptide loss in new-onset T1DM. However, whether TNF-α can prevent or delay T1DM onset has not been evaluated [126]. Thus, evaluation of new immunosuppressant with long-term efficacy and without β-cell toxicity combined with β-cell proliferation agent is fundamental to improving the overall outcomes of T1DM.

DMF is an immunosuppressant with anti-inflammatory and antioxidative properties that has also been shown to have protective effects on pancreatic β-cells [120,128]. DMF has been successfully used to treat multiple sclerosis (MS) and psoriasis. In the MS clinical trial, DMF treatment significantly decreased both T and B cell counts in MS patients [129]. Importantly, the effect of DMF differs based on the T cell subpopulation, and it has been shown that DMF increased CD4/CD8 and naive/memory T cells while reducing the frequency of T helper 1 (Th1) and Th17 inflammatory cells in DMF-treated MS patients [130]. Furthermore, DMF has also been shown to decrease follicular helper T cell, a subset of T cells critical for B cell activation to increase proliferation and antibody production [131]. In addition to altering T and B cell population, DMF has also been shown to alter activity of macrophages. Although yet to be studied in humans, animal models have demonstrated that DMF decreased M1(pro-inflammatory)/M2(anti-inflammatory) macrophage polarization and absolute number. DMF treatment of a mouse model of immune thrombocytopenia, an autoimmune disease characterized by immune mediated platelet destruction, demonstrated reduced number of CD68+ macrophages in the spleen, and in an in vitro study, DMF induced apoptosis of macrophages dose dependently. Lastly, in support of these findings, we previously reported that DMF significantly delayed the onset of T1DM in non-obese diabetic mice and reduced the onset of autoimmune DM. The insulitis score was significantly lower, and these results were accompanied by a significant reduction in serum level of proinflammatory cytokines and chemokines [132]. Thus, combination therapy with DMF is a novel approach that may augment the efficacy of proliferation agents by providing protection from autoreactive immune cell destruction.

## 6. Conclusions

In conclusion, in both T1DM and T2DM, pancreatic β-cells face several obstacles hampering their ability to regulate blood glucose levels. In contrast, in T1DM, β-cell destruction by autoreactive immune cells causes reduced β-cell mass; in T2DM, diabetogenic insults result in major changes in pathways (PI3K-AKT/PKB, Ras/Raf/ERK, cell cycle regulators) that impair the ability of β-cells to proliferate. Thus, there has been a focus on identifying therapeutic agents capable of inducing β-cell proliferation in human islets, most importantly, gastrin, DYRK1A, and GABA, as discussed earlier. While promising, further studies on combination therapy with proliferation agents must be conducted to develop a comprehensive treatment regimen for both T1DM and T2DM. Nrf2 activators for T2DM and DMF for T1DM have tremendous potential in fulfilling these roles to provide protection of newly proliferated β-cells. Evaluating other therapeutic agents that could be used in the combination therapy is an exciting avenue to explore.

## Data Availability

The data are contained within the article.

## Abbreviations

T1DM	Type 1 Diabetes
T2DM	Type 2 Diabetes
OGTT	Oral Glucose Tolerance Test
HOMA2	Homeostasis Model Assessment
iNOS	Nitric Oxide Synthase
PI3K—AKT/PKB	Phosphoinositide 3 Kinases-AKT/Protein Kinase B
FOXO1	Forkhead Box Protein O1
GSK3	Glycogen Synthase Kinase-3
mTOR	Mammalian Target Rapamycin
IRS2	Insulin Receptor Substrate 2
GCK	Glucokinase
ERK	Extracellular Signal-Regulated Kinase
MAPK	Mitogen-Activated Protein Kinase
MafA	MAF BZIP Transcription Factor A
PDX-1	Pancreatic and Duodenal Homeobox 1
GLUT2	Glucose Transporter 2
PCNA	Proliferating Cell Nuclear Antigen
CDK2	Cyclin-Dependent Kinase 2
NOD mouse	Non-Obese Diabetic Mouse
IPGTT	Intraperitoneal Glucose Tolerance Test
GLP1	Glucagon-Like Peptide 1
HbA1C	Hemoglobin A1C
NFAT	Nuclear Factor of Activated T-Cells
PRLR	Prolactin Receptor
PDGF	Platelet-Derived Growth Factor
WISP1	Wnt-Induced Signaling Protein 1
PPI	Proton Pump Inhibitors
EGF	Epidermal Growth Factor
INS	Insulin
NKX1.6	NKX Homeobox 1
NKX2.2	NK2 Homeobox 2
MNX1	Motor Neuron and Pancreas Homeobox 1
CCKBR	Dholecystokinin B Receptor
DYRK1A	Dual Specificity Tyrosine Phosphorylation Regulated Kinase 1A
IEQ	Islet Equivalent
GABA	γ-Aminobutyric Acid
CREB	cAMP Response Element Binding Protein
TGF β	Transforming Growth Factor β
STZ	Streptozotocin
NSG mouse	NOD Scid Gamma Mouse
Nrf2	Nuclear Factor Erythroid Factor 2-Related Factor 2
HO-1	Heme Oxygenase 1
G6PD	Glucose 6 Phosphate Dehydrogenase
TXNRD1	Thioredoxin Reductase 1
DMF	Dimethyl Fumarate
MS	Multiple Sclerosis
Th1	T-Helper 1
